# Characterization of Phytochemicals in Berry Fruit Wines Analyzed by Liquid Chromatography Coupled to Photodiode-Array Detection and Electrospray Ionization/Ion Trap Mass Spectrometry (LC-DAD-ESI-MS^n^) and Their Antioxidant and Antimicrobial Activity

**DOI:** 10.3390/foods9121783

**Published:** 2020-12-01

**Authors:** Agata Czyżowska, Agnieszka Wilkowska, Agnieszka Staszczak (Mianowska), Agnieszka Nowak

**Affiliations:** Institute of Fermentation Technology and Microbiology, Faculty of Biotechnology and Food Sciences, Lodz University of Technology, 171/173 Wolczanska Street, 90-924 Lodz, Poland; agnieszka.wilkowska@p.lodz.pl (A.W.); agnieszkamianowska@op.pl (A.S.); agnieszka.nowak@p.lodz.pl (A.N.)

**Keywords:** berries, fruit wines polyphenols identification, LC–MS^n^

## Abstract

Fruits are a valuable source of phytochemicals. However, there is little detailed information about the compounds contained in fruit wines. In this study, wines from six different berries were analyzed using HPLC-DAD-ESI-MS^n^. About 150 compounds were identified, including anthocyanins (34), hydroxycinnamic acids (12) and flavonols (36). Some of the compounds were identified for the first time in berry wines. The blackberry wines were found to contain the largest number of bioactive compounds (59). Elderberry wines where the richest source of polyphenols (over 1000 mg/L) and contained the largest amounts of all of the analyzed groups of compounds (hydroxycinnamic acids, anthocyanins and flavonols). The lowest concentration of polyphenols was observed in the wines made from cranberries and bilberries (less than 500 mg/L). The antioxidant activity was determined in relation to ABTS^+^, DPPH, and FRAP. The highest values were observed in the blackberry wines, and the lowest for the cranberry wines. The wines were analyzed to test their antimicrobial activity. Five of the six wines (with the exception of elderberry wine) inhibited *Bacillus cereus* growth and two (blackberry and cranberry wines) were active against *Listeria monocytogenes*.

## 1. Introduction

Fruits are known to be a valuable source of phytochemicals. However, there is little detailed information in the literature about the compounds contained in fruit wines [1,2,3,4,5,6,7,8,9]. Only fruit wines from strawberries and bilberries (*Vaccinium myrtillus* L.) have been studied intensively [10,11,12]. Behrend and Weber [10] analyzed the anthocyanins and tannins in bilberry wines fermented after different pretreatments and during ageing. Liquid chromatography–mass spectrometry (LC–MS) of the anthocyanins was performed using an LTQ-XL ion trap mass spectrometer connected to a UHPLC system via an electrospray ionization (ESI) interface. The anthocyanin profiles of all the wines were identical at the start of fermentation. During fermentation, considerable changes were noticed. The wines that had been subjected to prefermentative thermal treatment had an almost juice-like composition. The other wines displayed lower levels of arabinosides and galactosides. Polymeric pigments and pyranoanthocyanins were observed in all the wines. Liu et al. [12] analyzed bilberry wines by liquid chromatography using a diode array detector and electrospray ionization-quadrapole/time of flight hybrid mass spectrometry (ESI-QTOF-MS). They identified 42 nonanthocyanin compounds, including 22 phenolic acids, 15 flavonols and 5 flavan-3-ols. Hornedo-Ortega et al. [11] analyzed the anthocyanins in strawberry beverages. The anthocyanin fraction of the fermented strawberry wine was analyzed on an Amberlite XAD7HP column. Four anthocyanin compounds were identified with high accuracy for the first time in strawberry wines: pelargonidin-3-sambubioside, pelargonidin disaccharide (hexose + pentose) acylated with acetic acid, cyanidin-3-(6-acetyl)-glucoside, and pelargonidin 3-(6-succinyl)-arabinoside/3-(6-malonyl)-rhamnoside.

Papadopoulou et al. [13] demonstrated the antibacterial activity of the polyphenols in various white and red wines against strains of *Staphylococcus aureus* and *Escherichia coli.* Fruit wines, which are also a rich source of polyphenolic compounds and other bioactive compounds [2,3,4,5,6,7,8,9], may have similar antibacterial properties.

Polyphenols can also show antifungal activity, although it is much weaker [13,14]. Moreover, via various mechanisms, polyphenolic compounds limit the acquisition of resistance by microorganisms [15].

Red and purple fruits are rich sources of anthocyanins, which show bacteriostatic and bactericidal activity against many microorganisms (including *Staphylococcus* sp., *Klebsiella* sp., *Helicobacter* and *Bacillus*) [16,17]. Raspberries and cloudberries are rich sources of ellagitannins. These compounds are also found in strawberries, but in smaller amounts [17,18]. Quercetin is another compound found in fruits. It has been shown to increase the permeability of bacterial cell wall, which may, for example, increase the sensitivity of bacteria to antibiotics [19]. In many cases, mixtures of these compounds have been found to have stronger effects than any of their components separately. Of the various berries, raspberries and cloudberries (*Rubus chamaemorus*) are recognized as the best inhibitors of bacteria such as *Staphylococcus* spp., *Salmonella* spp., *Helicobacter pylori* and *Bacillus cereus* [20,21]. Raspberry juice has been reported to completely inhibit the growth of *Escherichia coli* in vitro [22].

Berries are also a rich source of vitamins A, C, and E. Ascorbic acid is found in a wide variety of fresh fruits [23]. In addition to having redox potential, it is also an excellent electron donor in biological systems [24]. Epidemiological and experimental evidence suggests that vitamin C can protect against the development of gastric cancer by several potential mechanisms: it reduces gastric mucosal oxidative stress, DNA damage, and gastric inflammation by scavenging ROS (reactive oxygen species); it inhibits gastric nitrosation and the formation of N-nitroso compounds by reducing nitrous acid to nitric oxide and producing dehydroascorbic acid in the stomach; it enhances host immunologic functions; it has a direct effect on *Helicobacter pylori* growth and virulence; it inhibits gastric cell proliferation and induces apoptosis [25]. The content of vitamin C in berry fruits can be influenced by numerous factors, including the species, variety, weather conditions, ripeness, and region [23]. Vitamin stability can be affected by various technological practices used during the processing of food, namely changes in temperature (e.g., thermal treatments) and oxygen levels [24]. The concentration of vitamins decreases during winemaking (fermentation and ageing) [26].

In this study, we characterize and quantify the bioactive compounds in wines made from six different berries, using HPLC-DAD-ESI-MS^n^. We also investigate whether the fruit wines may be considered a source of antimicrobial agents against pathogenic microorganisms. The fruit wines were tested against both pathogenic Gram-negative bacteria (*E. coli* and *Salmonella* Enteritidis), which can cause foodborne and waterborne outbreaks of gastrointestinal tract infections, and pathogenic Gram-positive bacteria (*B. cereus*, *L. monocytogenes* and *S. aureus*), which can cause food poisoning and toxic symptoms in humans. *Candida albicans* ATCC 10231, was used as a reference strain for the analysis of antifungal action [27]. Some of these microbes can also colonize oral human cavities [28,29,30,31,32]. Studies suggest that between 94% and 100% of healthy adults have oral colonization with *Staphylococcus* spp. [30] and oral carriage of *S. aureus* ranges from 24% to 36% [31].

## 2. Materials and Methods

### 2.1. Reagents and Standards

ABTS^+•^ (2,2′-azinobis 3-ethylbenzothiazoline-6-sulfonic acid), potassium persulphate, FeCl_3_, TPTZ (2,4,6-Tris (2-pyridyl-S-triazine), DPPH^+•^ (2,2-diphenyl-1-picrylhydrazyl) and methanol were purchased from Sigma (Poznań, Poland). Formic acid and HPLC-grade acetonitrile were sourced from J.T. Baker (Witko, Poland). Anthocyanin standards were produced by Extrasynthese (Genay, France) and PhytoLab (Vestenbergsgreuth, Germany). Available standards of other polyphenols were purchased from Sigma (Poznań, Poland) and Extrasynthese (Genay, France). HPLC-grade water was obtained using an Aquinity E60 Lifescience TI system (membraPure GmbH, Bodenheim, Germany).

### 2.2. Wine Preparation

Fruit wines were prepared according to the Polish Law of 12 May 2011 ‘On the production and bottling of wine products, trade and organization of the wine market’. Six wine types were made from the following berries: bilberry (common bilberry) (*Vaccinium myrtillus* L.)—BB; blackberry (*Rubus* L.)—B; cranberry (*Vaccinium macrocarpon* Aiton)—C; elderberry (*Sambucus nigra* L.)—E; raspberry (*Rubus idaeus* L.)—R; and strawberry *(Fragaria × ananassa*)—S (Kent variety).

Fresh blackberry, cranberry, raspberry and strawberry fruits (about 10 kg of each species) were purchased from local retailers between June and October, depending on the availability. Bilberry fruits were collected in the region of Belchatow (51°21′ N, 19°21′ E) and elderberry fruits in Pabianice (51°39′ N, 19°21′ E). The elderberry stalks were removed. The fruits were then heat treated (85 °C, 5 min) to inactivate polyphenol oxidase-type enzymes. The blueberry, elderberry and cranberry pulps were cooled to 50 °C and treated with pectinolytic enzyme (Rohapect 10 L, AB Enzymes GmbH, Darmstadt Germany, AKE, Pabianice, Poland) at a dose of 0.5 g/kg of fruits. They were then pressed using a hydraulic press.

Fermentation was performed at 25 °C using BCS103 wine yeast (Fermentis, LeMag, Żyrardów, Poland) at a dose of 0.2 g/L. Once fermentation was complete, the wine was racked and poured into bottles. All the wines were aged for around 5 months. The wines were then subjected to basic analysis (alcohol, extract, sugar, acidity). The wines were dealcoholated and tested for their antimicrobial activity.

### 2.3. Preparation of Dealcoholated Red Wines (DRW)

To remove the alcohol from the wines, an equal volume of distilled water was added to a given volume of wine and then concentrated to the original volume (38 mbar, 35 °C, 140–180 rev/min). The solutions were concentrated on a Büchi vacuum evaporator—Rotavapor R-215 (Büchi Labortechnik AG, Flawil, Switzerland).

### 2.4. Analysis of Organic Acids, Sugars and Alcohols

Organic acids, glucose, fructose and alcohols (ethanol and glycerol) were analyzed using a Finnigan Surveyor HPLC system (Thermo Fisher Scientific Inc., Waltham, MA, USA), according to the method described by Czyżowska et al. [33].

### 2.5. Total Phenolic Content (TPC) Assay

Total phenolic content (TPC) was determined using the Folin–Ciocalteu reaction with gallic acid as a standard. To a test tube were added 0.1 mL of the 5-fold diluted sample, 0.2 mL of Folin–Ciocalteu reagent, 1 mL of 20% sodium carbonate and 2 mL of distilled water. In the control, 0.1 mL of distilled water was added instead of the test solution. The samples were mixed and incubated for 1 h at room temperature, in the absence of light. After incubation, the absorbance was measured at a wavelength of λ = 765 nm (Cecil CE 2041, Cecil Instruments Limited, Cambridge, UK).

### 2.6. Analysis of Antioxidant Capacity

#### 2.6.1. ABTS Radical-Scavenging System

Radical scavenging activity against ABTS^+•^ was determined based on the method described by Rivero-Pérez et al. [34] with slight modifications. The wine solution (0.2 mL) was mixed with 4 mL of ABTS reagent and incubated for 15 min. The results were expressed as mM of Trolox equivalents, using linear calibration obtained with different concentrations of Trolox.

#### 2.6.2. DPPH Radical-Scavenging System

The method described by Fogliano et al. [35] was applied with slight modifications. Wine solution (0.2 mL) was mixed with 4 mL of DPPH^+•^ reagent (65 μM) and incubated for 30 min. Absorbance was measured at 515 nm. The results were expressed as mM of Trolox equivalents on the relevant calibration curve.

#### 2.6.3. FRAP Method

The method described by Rivero-Pérez et al. [34] was used with slight modifications. For 30 min, 2.9 mL of the reactive mixture was incubated with 50 μL of the sample. Absorbance was measured at 595 nm. The results were expressed as mM of Trolox equivalents on the relevant calibration curve.

### 2.7. LC–MS^n^ Identification of Wines Compounds

Qualitative analysis of the bioactive compounds in the berry wines was conducted using an HPLC coupled on-line with an MS LTQ Velos mass spectrometer (ThermoScientific, Waltham, MA, USA), following the method described by Efenberger-Szmechtyk et al. [36]. Separation was carried out using a Hypersil Gold column (150 × 2.1, particle size 1.9 μm) (ThermoScientific, Waltham, MA, USA). The column was thermostated at 45 °C. For the anthocyanins, 2.5% formic acid solution (phase A) and 95% acetonitrile (phase B) were used as eluents with a flow rate of 220 µL/min and an injection volume of 10 µL. For other compounds, the mobile phase consisted of solvent A (1 mL formic acid in 1 L of deionized water) and solvent B (95% acetonitryl). The separation was carried out with the following gradients: in the first 8 min, a linear gradient from 96% to 85% phase A; 8–12 min linear gradient from 85% to 82% phase A; 12–40 min linear gradient from 82% to 60% phase A; 40–44 min linear gradient from 60% to 50% phase A; 44–47 min linear gradient from 60% to 50% phase A; 47–49 min linear gradient from 50% to 96% phase A, followed by column recalibration.

Spectrometry was performed with a capillary voltage of 4 kV and collision energy of 20 V. The desolvation temperature was 280 °C and the source temperature was 350 °C.

Detection of anthocyanins was carried out in positive ion mode, whereas the other compounds were detected in the negative ion mode in the range of *m*/*z* from 100 to 1200. The compounds were identified based on a comparison of the maximum absorption spectra of UV radiation. The molecular weight was determined on the basis of the mass to charge ratio. Retention times and fragmentation spectra were compared with the available standards and literature data.

### 2.8. HPLC Analysis of Polyphenols

Prior to analysis, the samples were filtered through a 0.45 μm membrane and injected into the HPLC system. HPLC-PDA analyses were performed using a Finnigan Surveyor equipped with an autosampler, a diode array detector Finnigan Surveyor-PDA Plus (Thermo FisherScientific Inc., Waltham, MA, USA) and ChromQuest 5.0 chromatography software (Thermo FisherScientific Inc., Waltham, MA, USA). The separation conditions were as described by Efenberger-Szmechtyk et al. [36]. The calibration curves were established using standards for chlorogenic acid, quercetin-glucoside and cyanidin-glucoside to quantify polyphenols at 320, 360 and 520 nm, respectively.

### 2.9. Analysis of Antimicrobial Activity in Dealcoholated Red Wines (DRW)

The biological materials used for the antimicrobial tests were strains of bacteria and yeasts: *Bacillus cereus* ŁOCK 0807, *Escherichia coli* ATTC 10536, *Listeria monocytogenes* ATTC 13932, *Salmonella Enteritidis* ATTC 13076, *Staphylococcus aureus* ATCC 6538 and *Candida albicans* ATCC 10231.

#### Antimicrobial Assay

The agar well diffusion method was used to verify whether the DRW affected the growth of the microorganisms [9]. A TSB (Trypticase soy broth) medium was used to activate cultures of bacteria following storage in the CRYOBANK^TM^ system and YPD (Yeast Extract–Peptone–Dextrose) medium was used to activate the yeasts. After 24 h, the cultures were submitted for further analysis. Standardized inocula of the tested microorganisms were incubated in TSA (Trypticase soy agar) or YPD, depending on the groups of microorganisms. Next, wells with a diameter of 9 mm were punched aseptically with a sterile cork borer. To each well was added 120 μL of DRW. The samples were incubated at 37 °C for bacteria and 30 °C for yeast. After incubation, the inhibition zones were measured.

### 2.10. Statistical Analysis

Mean values, standard deviations and the occurrence of statistically significant differences were determined using STATISTICA 10 PL software (StatSoft, Krakow, Poland). The ANOVA test was used, assuming a significance level of 0.05.

## 3. Results and Discussion

### 3.1. Sugars, Organic Acids and Alcohols

The alcohol content of the wines ranged from 7.22% to 14.59% (*v*/*v*, Table 1). The lowest alcohol content was detected in the cranberry wines and the highest in the elderberry wines. The wines with the highest alcohol contents had low or trace amounts of glucose.

Glycerol was the main by-product in wines obtained in our study. Its content was between 4.95 and 10.81 g/L. It was also the main compound in five of the eight raspberry wines investigated by Duarte et al. [37], with contents ranging from 4.6 to 10.2 g/L. In subsequent research by Duarte et al. [38], glycerol was again the main compound in 11 out of the 16 studied raspberry wines. The glycerol contents were similar to those reported in their previous study, in the range of 4.45–10.11 g/L.

Acidity is one of the most important parameters in wine. In grape wines, it is mainly associated with organic acids such as tartaric, malic, acetic and lactic acids. Citric acid can have a significant effect on fruit wines, due to its high concentration in the raw material (for example in raspberries and blackberries). Malic acid also contributes to the acidity of fruit wine. Lactic and succinic acids are produced during fermentation. In our study, these two compounds were produced in amounts lower than 1 g/L. The predominant volatile acid is acetic acid, often expressed as the wine-quality parameter and known as volatile acidity. It is always formed during alcoholic fermentation. Higher concentrations of acetic acid can affect the organoleptic properties of wine. The acetic acid content was under the limit of quantification in the elderberry, raspberry, and strawberry wines. The highest content was observed in the bilberry wines.

Duarte et al. found a high content of succinic acid in raspberry wines [37]. In only one sample was it below 5.0 (2.8 g/L), whereas, in the others, it was from 5.6 to 7.1 g/L [38]. This is almost ten times higher than the levels of succinic acid found in our raspberry wines. Duarte et al. [38] also reported higher acetic acid contents, from 0.7 to 2.3 g/L. Most of the wines analyzed had no glucose.

In our elderberry wines, citric acid was the main acid, at concentrations of around 2.40 g/L. Malic acid was present in the next highest quantities (1.15 g/L). This is consistent with results reported by Veberic et al. [39] for two cultivars and three selections of black elderberries from Slovenia. These authors found four acids (citric, malic, shikimic and fumaric) in the fruit.

Citric acid was present in the largest quantity, the content of malic acid was around three times smaller, and shikimic and fumaric acids composed on average 10% of the total.

Four of the six tested wines showed the presence of ascorbic acid. The concentration ranged from 0.03 to 0.08 g/L, for cranberry and bilberry wines, respectively. The low levels of vitamins in grape wines may explain the lack of relevant studies regarding the content of vitamins in fruit wines. Grape wines are reported to contain some B vitamins and very small amounts of vitamin C and fat-soluble vitamins [26]. Vitamin C can be destroyed during processing and storage.

### 3.2. Total Content of Polyphenols and Antioxidant Activity of Fruit Wines

Of the wines investigated in our study, wines made from elderberry fruits showed the highest content of total polyphenols (1480.47 mg/L) (Table 2). The content of total polyphenols in the remaining wines ranged from 408.03 to 759.42 mg GAE/L. Low concentrations of polyphenols were observed in wines from cranberries and bilberries (below 500 mg/L).

In a study by Rupasinghe and Clegg [6], elderberry wines from Canada were found to contain 1753 mg GAE/L. Aged elderberry wines from Slovenia analyzed by Schmitzer et al. [7] contained 1584.99 mg/L of total polyphenols. The pitchings (musts) were prepared in a similar manner to our elderberry wines, and the results are similar.

Most studies that have measured the content of total polyphenols in blackberry and bilberry wines used commercial products, so the methods of preparation are not known. Blackberry and bilberry wines from Illinois have been reported as having total polyphenol contents ranging from 188 mg GAE/L to 1115 mg GAE/L [40]. These levels are comparable to those for the blackberry and bilberry wines in our study. The blackberry and bilberry wines investigated by Kalkan Yildrim [41] had slightly higher concentrations of polyphenols. The blackberry wines studied by Ortiz et al. [42] contained from 1122 to 1400 mg/L of total polyphenols (depending on the pectinolytic enzyme used). The blackberry wines analyzed by Ljevar et al. [43] contained from 1055 to 2704 mg/L. The highest contents of total polyphenols in blackberry wines have been reported by Mudnic et al. [44] (1697–2789 mg/L) and Mitic et al. [45] (1608–2836 mg/L).

The total polyphenol contents reported by Mitic et al. [45] and Ljevar et al. [43] for raspberry wines from Serbia and Croatia, respectively, were up to three-fold higher than those found in the raspberry wines in our study (1052–1490 and 1199–1840 mg/L, respectively). However, the preparation method again was different [46]. The raspberry and cranberry wines investigated by Rupasinghe and Clegg [6] contained 977 and 971 mg/L of total polyphenols, respectively.

The total content of polyphenols in our strawberry wines was almost three times lower than in wines obtained by Cakar et al. [1].

One method is usually insufficient to evaluate the antioxidant activity of a complex substance. In our study, three different assays were used to study the antioxidant properties of the wines. The antioxidant activity was determined in relation to ABTS^+^, DPPH and FRAP. Significant differences between ABTS and DPPH radicals were found in the examined wines, but similar trends were observed in different assays (FRAP, ABTS, and DPPH). The highest values were observed in blackberry wines, and the lowest for the cranberry wines (Table 2). As in a study by Ljevar et al. [43], the blackberry wines in our research also showed the highest antioxidant activity. The elderberry wines, despite having the highest polyphenol content, did not show high antioxidant activity. Heinonen et al. [5] evaluated the antioxidant activity of over 44 different fruit wines, mainly berry wines. The results showed that the total phenolic content did not correlate with the antioxidant activity. On the other hand, some studies have confirmed a strong positive correlation between the total antioxidant activity in fruit wines and total phenolics [6,46].

A study by Gao et al. [47] investigating the contribution of three different antioxidant fractions using an ABTS assay showed the total antioxidant capacity of phenolics, ascorbic acid, and lipophilic compounds to be slightly lower than those of crude extracts. The phenolic fraction made a major contribution to the total activity (about 75%), followed by ascorbic acid (around 17%). According to Brand-Williams et al. [48], ascorbic acid is one of the fastest reacting antioxidants. Observing changes in the phenolic profile during the winemaking process, Lingua et al. [49] noted that anthocyanins were the most important phenols in the wine samples. Most berry wines are rich in anthocyanins, but they react poorly in the Folin–Ciocalteu test, giving a poor correlation [50]. In general, different compounds were selected to correlate with the different in vitro assays. Depending on the chemical structure of the compounds and the mechanisms involved (hydrogen atom transfer, single electron transfer, reducing power, and metal chelation, among others), they react differently in various vitro assays [51].

### 3.3. Polyphenols in Wines

As there is no extensive literature on the subject, qualitative and quantitative analysis of the composition of polyphenols in the fruit wines was based mainly on the data concerning the juices and fruits from which the wines originated.

#### 3.3.1. Anthocyanins

Cyanidin 3-glucoside was present in all the samples (Table 3; Figures S1, S4, S7, S10, S13 and S16). The highest level of cyanidin-glucoside was found in the blackberry wines (30.26 mg/L). Cyanidin 3-galactoside was found in four of investigated wines. Traces of cyanidin 3-rutinoside were found in the blackberry and raspberry wines.

Cyanidin-sophoroside and cyanidin 3-(2G-glucosylrutinoside) were found in the raspberry wines, with Cy-soph as the main compound (Figure S13). Cyanidin 3-sambubioside and cyanidin 3-sambubioside-5-glucoside were present only in the elderberry wines, in quantities of 46.51 mg/L and 23.07 mg/L, respectively (Figure S10).

Delphinidin derivatives were only present in the bilberry wines (Figure S1). Malvidin derivatives were also detected only in the bilberry wines, whereas pelargonidin derivatives were found in the wines made from blackberries and strawberries (Figures S4 and S16). Pelargonidin 3-glucoside was the main anthocyanin found in the strawberry wines. Carboxypyranopelargonidin-glucoside (CP Pg-glc) was also found in the strawberry wines. This compound had been identified previously in strawberries [52] and in strawberry-fermented products [11,53].

Peonidins were found in the bilberry, blackberry and cranberry wines. Petunidins were found only in the bilberry wines.

The highest concentrations of anthocyanins were found in the elderberry wines (76.33 mg/L). The concentrations of anthocyanins in the blackberry wines were in excess of 50 mg/L.

The main compound present in elderberry wines was cyanidin 3-sambubioside (46.51 mg/L), and cyanidin 3-sambubioside-5-glucoside was present in the next largest quantities.

Our results differ significantly from those reported by Schmitzer et al. [7]. In their study of elderberry wines, cyanidin 3-glucoside was present at the highest concentrations in mature wine (20.88 mg/L), whereas, in our study, the concentration of this compound was 6.19 mg/L. According to Schmitzer et al., cyanidin 3,5-diglucoside was present at a slightly lower concentration (18.49 mg/L), whereas, in our study, this compound was not found in the elderberry wines. Cyanidin 3,5-diglucoside and cyanidin 3-sambubioside-5-glucoside have similar retention times and coelution sometimes occurs [54]. However, no compound with a mass characteristic of cyanidin 3,5-diglucoside was found during our investigation. The sum of the concentrations of anthocyanins observed by Schmitzer et al. [7] based on HPLC analysis was approximately 50% higher than that in our elderberry wines.

The dominant compound in the blackberry wines we studied was cyanidin 3-glucoside, at a concentration of 30.26 mg/L. In domestic wines obtained by Mitic et al. [45], the content of cyanidin 3-glucoside was around 10 times higher. Cyanidin xyloside was present in the next highest concentration, which was again much higher than in the blackberry wines in our study. Cyanidin 3-rutinoside was the main compound in the blackberry wines analyzed by Ljevar et al. [43]. Only small amounts of this compound were found in the blackberry wines we studied. Relatively large amounts of cyanidin 3-glucoside acylated with malonic acid were detected in our study, at a concentration approximately two-fold lower than those for cyanidin 3-glucoside. This compound has been identified previously as occurring in blackberries [54,55,56,57,58] but was identified here for the first time in blackberry wine.

Compared to those analyzed by Hornedo-Ortega et al. [53], the strawberry wines in our study contained much lower levels of both pelargonidin 3-glucoside and pelargonidin 3-rutinoside. However, the wines studied by Hornedo-Ortega et al. were analyzed immediately following the fermentation process, whereas ours were tested after 5 months of aging. Other authors have noted a 63–85% reduction in these compounds during the fermentation and storage [59,60]. Relatively large amounts of one acylated derivative and one diglucoside derivative of pelargonidin were found in our wines. Hornedo-Ortega et al. [53] showed that fermentation significantly increases the levels of diglucoside in strawberry wines (2.5- and 6.2-fold for 2012 and 2013 vintages, respectively).

All of the wines in our study were prepared using thermal treatment. As observed by Behrends and Weber [10], pre-fermentative heat treatment influences the characteristics of wine. This was confirmed in our previous research [2,3]. The bilberry wines investigated by Behrends and Weber [10] had almost the same anthocyanin profiles as juices when pre-fermentative heat treatment was applied. With warm treatment (70 °C), the ratio of glucosides to galactosides and arabinosides was 47.9:32.1:20. In the bilberry wines we studied, the ratio was 58.9:32:9.1. Some of the fruits in our study (including bilberries) were also treated with pectinase. As observed by Buchert et al. [61], enzyme-assisted bilberry juice production leads to greater losses of galactosides.

#### 3.3.2. Phenolic Acids

The largest quantities of hydroxycinnamic acids were found in elderberry wines (150.79 mg/L). The lowest levels were observed in the strawberry wines (13.43 mg/L) (Table 4). None of the acids were present in all of the wines (Figures S2, S5, S8, S11, S14 and S17).

The main acids found in the elderberry wines were neochlorogenic, chlorogenic and caffeic acids (Figure S11). Schmitzer et al. [7] analyzed only neochlorogenic and chlorogenic acids. The neochlorogenic acid content was higher in our samples. It is difficult to compare results for chlorogenic acid. During chromatographic analysis (HPLC), chlorogenic acid probably coeluted with caffeic acid and one large peak was observed. Using a mass spectrometer, a mass of 179 (typical for caffeic acid) proved dominant. Caffeic acid hexoside (341 *m*/*z*), *p*-coumaric acid (163 *m*/*z*) and p-coumaric acid derivative (525 *m*/*z*) were also found. Caffeic acid and its derivative, caffeic acid hexoside, were the main acids in the bilberry wines, accounting for almost 75% of the total acid content (Figure S2). Caffeic acid was also the main compound in the raspberry wines. The dominant acids in the blackberry wines (around 85%) and strawberry wines (around 47%) were p-coumaroylhexosides. Cakar et al. [1] found small amounts (4.16–2.83 µg/mL) of p-coumaric acid in strawberry wines, which was about 10% of the total phenolic acid content. These authors identified chlorogenic acid chlorogenic acid as a main compound (290–335 µg/mL) followed by the caffeic acid. No chlorogenic acid was identified in any of the 90 strawberry varieties investigated by Nowicka et al. [62]. A compound with very similar MS (*m*/*z* 355) was identified as 1-*O*-trans-cinnamoyl-glucose. This compound had the highest content in almost all investigated varieties, followed by *p*-coumaroyl-glucosides. Nowicka et al. [62] also identified 1-*O*-feruloylglucose. In our investigated wines, 5-hydroxyferuloylhexoside was identified. This compound probably formed during fermentation.

#### 3.3.3. Flavonols

Flavonols were present in relatively low concentrations compared to the other groups of studied compounds, from 0.87 to 9.27 mg/L (Table 5). The lowest concentrations occurred in wines made from raspberries, strawberries, bilberries and blackberries (0.87–1.81 mg/L), and the highest in wines made from elderberries (9.27 mg/L).

In terms of qualitative composition, more than 80% of all identified flavonols in the elderberry wines were quercetin and its derivatives (Figure S12). Myricetin and its derivatives were found in the cranberry and bilberry wines (Figures S3 and S9), while trace amounts of myricetin malonylglucoside were found in strawberry wines. Quercetin 3-rutinoside was the main flavonol in the elderberry wines, comprising 56% of the total for this group of compounds. Flavonols were found to dominate in the elderberry wines studied by Schmitzer et al. [7], at concentrations around 10 times higher than in our wines, with the highest concentrations occurring in young wines. The same authors detected quercetin rutinoside and glucoside in the highest and second-highest concentrations. Kaempferol-rutinoside was present in the lowest quantity, at a concentration of 1.1 mg/L, whereas, in our elderberry wines, the concentration was 0.22 mg/L. The elderberry wines in our study contained more than 1 mg/L of quercetin. Fruit wines made from cherries, blackberries and raspberries were found by Mitic et al. [45] to contain derivatives of quercetin and kaempferol. The same authors reported the presence of kaempferol derivatives in blackberry wines, at levels of 0.30–0.85 mg/L. However, we did not detect these compounds in our blackberry wines (Figure S6). The content of quercetin derivatives in the raspberry wines studied by Mitic et al. [45] ranged from 0.98 to 1.80 mg/L, compared with only 0.48 mg/L in our study. We also found kaempferol derivatives in raspberry wines (Figure S15), at a concentration of 0.39 mg/L. The strawberry wines investigated by Cakar et al. [1] contained three compounds from the group of flavonols: quercetin, quercetin 3-rutinoside (rutin) and kaempferol. Only small amounts of quercetin were found in our strawberry wines, less than 10% of the total Q-derivatives content. We did not identify Q-rut, but we found significant amounts of Q-glucoside and Q-glucuronide (Figure S18). We were unable to identify kaempferol in the strawberry wines, but we did find its derivatives, K-glucoside and K-glucuronide, as well as traces of K-pentoside and dihydroK-glucoside.

#### 3.3.4. Other Bioactive Compounds

Other bioactive compounds were tentatively identified using LC–MS^n^ (Table 6). Sixteen acids were found, including hydroxybenzoic acids. Cinnamic acid was identified in all the samples. Shikimic acid was found in all of the wines except cranberry wine. In the blackberry wines, two forms of abscisic acid were identified: abscisic acid d-glucopyranosyl ester (ABA-GE) and ursolic acid.

Ellagic acid was identified in five of the six wines in our study, but its derivatives were found mainly in the wines made from blackberries (five compounds), strawberries (three compounds) and raspberries (two compounds). Of the twelve ellagitannins we identified, as many as ten were found in the blackberry wines. Five were found in the strawberry wines and four were identified in the raspberry wines. Three gallic acid derivatives were identified: one in the bilberry wines, one in the cranberry wines and one in the strawberry wines. Procyanidins are another important group of polyphenols. In total, 20 compounds from this group were identified. Of these, 14 were found in the cranberry wines, which were the richest and most diverse source of procyanidins.

Seven compounds from this group were identified in the strawberry wines, including two afzelechin-catechin derivatives.

Cakar et al. [1] identified hydroxybenzoic acids in strawberry wines. Gallic acid had the highest concentration, followed consecutively by p-hydroxybenzoic, protocatechuic and vanillic acids. We identified two of these hydroxybenzoic acids in our strawberry wines (Table 6). We also found two derivatives of: protocatechuic acid hexoside and 1-*O*-protocatechuylhexoside. Vanillic acid was not found.

Cakar et al. also found significant amounts of ellagic acid in their strawberry wines. We identified this compound in five wines (was not present in cranberry wines) (Table 6).

### 3.4. Effect of Dealcoholated Fruit Wines on Microbial Growth

We studied the effects of the compounds present in the dealcoholated fruit wines on the growth of various microorganisms (Table 7). The berry wines had no inhibitory effect on the growth of *Salmonella* Enteritidis, *Staphylococcus aureus* bacteria and *Candida albicans* yeast. The only growth inhibitors for *Escherichia coli* ATCC 1053 were bioactive compounds found in the strawberry wines. The resulting zones of inhibition were 2.67 mm. The strain *Bacillus cereus* ŁOCK O807 was the most susceptible to the effects of the wines. Its growth was inhibited by the compounds in five of the wines. Only the elderberry wines had no effect on the growth of this strain. The most extensive inhibition zones resulted from the impact of raspberry wines.

Growth of *Listeria monocytogenes* ATCC 13932 was inhibited by the bioactive compounds present in two of the wines. The cranberry and blackberry wines had an inhibitory effect on the growth of these bacteria, with a zones of 2.33 and 1.00 mm, respectively.

The elderberry wines did not inhibit the growth of any of the microorganism, despite having the highest total polyphenol concentration and the highest content of anthocyanins, which some authors consider to be one of the main providers of antimicrobial properties [17,18]. The growth of *Bacillus cereus* was inhibited to the greatest extent by the wine made from raspberries. These fruits are a rich source of ellagitannins, which have strong antimicrobial activity. In the raspberry wines, we identified Sanguiin H2, H6 and Sanguiin H10 isomers (Table 6). Other compounds from this group (HHDP glucosides and their derivatives) were identified in the blackberry and strawberry wines. The cranberry wines did not contain ellagitannins, but a wide range of procyanidins were identified, including type A. Proanthocyanidin extracts of cranberries investigated by Kylli et al. [63] showed strong antimicrobial effects against *Staphylococcus aureus*, whereas they had no effect on other bacterial strains such as *Salmonella* Typhimurium and *Escherichia coli*. Phenolic extracts of lingonberry and cranberry had an antibacterial effect on Gram-positive pathogens including *Staphylococcus*, *Bacillus* and *Clostridium*, but only had weak or no antimicrobial activity on Gram-negative strains of *Salmonella*. However, *Listeria monocytogenes* was not inhibited by by either the lingonberry extract or cranberry extract [21,64].

Based on the results of these preliminary studies on the dealcoholated red berry wines, we see the possibility of using them as a food additive, improving safety and extending shelf life. As previously mentioned, some of the microorganisms tested may already be found in the oral cavity, so the wines could have an impact at this stage. Regarding the upper respiratory tract, oral invasion in immunosuppressed patients may be more frequent than previously documented, as the oral cavity can be colonized by *B. cereus* either by inhaling spores or by eating food contaminated with *B. cereus* [32]. Foci can occur when bacteria become trapped in the furrows in the oral cavity, where they grow and release toxins that spread to adjacent tissues and other parts of the body. However, further studies are necessary to investigate the action of the wines against pathogens in the human gastrointestinal tract.

## 4. Conclusions

In this study, about 150 compounds were identified in berry wines, including anthocyanins (34), hydroxycinnamic acids (12) and flavonols (36). Some of these compounds were identified for the first time in berry wines. The largest number of bioactive compounds was identified in the blackberry wines (59 compounds). All of the wines were rich in polyphenols. Elderberry wines were the richest source of polyphenols (over 1000 mg/L) and contained the largest amounts of all of analyzed groups of compounds (hydroxycinnamic acids, anthocyanins and flavonols). The lowest concentrations of polyphenols were found in wines made from cranberries and bilberries (below 500 mg/L). The dealcoholated berry wines were found to inhibit *Bacillus cereus* growth. Elderberry wines, despite their high content of polyphenols, did not show antimicrobial properties against the tested microorganisms. Antimicrobial properties may be affected by the combination and proportions of active compounds, and not only by the individual compounds. Our results show that berry fruit wines could provide biologically active compounds and at the same time protect against pathogens.

## Figures and Tables

**Table 1:** Organic acids, sugars, glycerol (g/L) and ethanol (*v*/*v* in the investigated wines).

	BB	B	C	E	R	S
citric acid	1.17 ± 0.15 ^a^	4.53 ± 0.35 ^d^	1.95 ± 0.09 ^b^	2.45 ± 0.08 ^c^	8.74 ± 0.65 ^f^	5.03 ± 0.15 ^e^
malic acid	0.65 ± 0.05 ^b^	2.03 ± 0.09 ^f^	1.63 ± 0.07 ^e^	1.15 ± 0.06 ^d^	0.44 ± 0.02 ^a^	0.95 ± 0.03 ^c^
succinic acid	0.71 ± 0.05 ^b^	0.99 ± 0.08 ^c^	0.76 ± 0.05 ^b^	0.92 ± 0.06 ^c^	0.62 ± 0.03 ^a^	0.56 ± 0.03 ^a^
lactic acid	0.28 ± 0.02 ^b^	0.02 ± 0.00 ^a^	0.02 ± 0.00 ^a^	0.54 ± 0.02 ^c^	0.02 ± 0.00 ^a^	0.02 ± 0.00 ^a^
acetic acid	0.50 ± 0.03 ^c^	0.24 ± 0.02 ^b^	0.09 ± 0.01 ^a^	uLOQ	uLOQ	uLOQ
ascorbic acid	0.08 ± 0.01 ^c^	0.04 ± 0.00 ^b^	0.03 ± 0.00 ^a^	uLOQ	0.07 ± 0.00 ^c^	uLOQ
glucose	uLOQ	uLOQ	43.43 ± 2.01 ^c^	0.04 ± 0.00 ^a^	0.10 ± 0.01 ^b^	uLOQ
fructose	0.47 ± 0.03 ^a^	0.68 ± 0.05 ^b^	29.06 ± 1.98 ^e^	0.67 ± 0.05 ^b^	0.85 ± 0.06 ^c^	1.62 ± 0.08 ^d^
glycerol	8.58 ± 0.65 ^c^	10.81 ± 0.77 ^d^	4.95 ± 0.32 ^a^	8.03 ± 0.63 ^bc^	5.41 ± 0.35 ^a^	6.96 ± 0.54 ^b^
ethanol	12.86 ± 1.01 ^c^	14.39 ± 1.06 ^d^	7.22 ± 0.56 ^a^	14.59 ± 0.99 ^d^	8.43 ± 0.65 ^b^	11.32 ± 0.87 ^c^

BB—bilberry wine; B—blackberry wine; C—cranberry wine; E—elderberry wine; R—raspberry wine; S—strawberry wine. uLOQ—uder limit of quantification; Different letters in rows indicate a significant difference (*p* < 0.05).

**Table 2:** Total polyphenols (mg/L) and antioxidant activity (mM of Trolox equivalents) of the investigated wines.

	BB	B	C	E	R	S
TP	466.82 ± 40.03 ^a^	759.42 ± 52.30 ^c^	408.03 ± 38.75 ^a^	1480.47 ± 103.55 ^d^	566.75 ± 43.02 ^b^	525.60 ± 50.74 ^b^
ABTS	4.29 ± 0.34 ^c^	5.84 ± 0.34 ^d^	3.03 ± 0.19 ^a^	4.22 ± 0.28 ^c^	3.49 ± 0.23 ^b^	3.40 ± 0.18 ^a^
DPPH	2.47 ± 0.19 ^c^	2.55 ± 0.16 ^d^	1.12 ± 0.07 ^a^	1.87 ± 0.09 ^b^	1.75 ± 0.07 ^b^	1.66 ± 0.12 ^b^
FRAP	5.07 ± 0.23 ^c^	6.45 ± 0.45 ^d^	3.32 ± 0.25 ^a^	5.07 ± 0.39 ^c^	4.42 ± 0.36 ^b^	3.47 ± 0.22 ^a^

BB—bilberry wine; B—blackberry wine; C—cranberry wine; E—elderberry wine; R—raspberry wine; S—strawberry wine; tr—traces; TP—total polyphenols; ABTS—2,2-azino-bis-3-ethylbenzothiazoline-6-sulfonic acid; DPPH—2,2-diphenyl-1-picrylhydrazyl; FRAP—Ferric reducing antioxidant power. Different letters in rows indicate a significant difference (*p* < 0.05).

**Table 3:** Content of anthocyanins in the investigated wines (mg/L).

Compound	[M + H]^+^ *m*/*z*	MS^2^ *m*/*z*	BB	B	C	E	R	S
Cy-gal	449	287	4.11 ± 0.32	1.11 ± 0.08	0.65 ± 0.05	-	-	0.32 ± 0.03
Cy-glc	449	287	LOQ	30.26 ± 2.89	0.29 ± 0.02	6.19 ± 0.57	3.29 ± 0.28	0.30 ± 0.03
Cy-ara	419	287	-	-	1.19 ± 0.09	-	-	-
Cy-xyl	419	287	-	1.34 ± 0.09	-	-	-	-
Cy-rut	595	287	-	LOQ	-	-	LOQ	-
Cy-soph	611	287	-	-	-	-	14.98 ± 1.32	-
Cy-3(2glc) rut	757	287	-	-	-	-	1.53 ± 0.11	-
Cy-3mal-glc	535	287	-	1.32 ± 0.12	-	-	-	-
Cy-6mal-glc	535	287	-	13.19 ± 1.09	-	-	-	-
Cy-sam	579	537, 357	-	-	-	46.51 ± 4.34	-	-
Cy-sam-5-glc	744	287	-	-	-	23.07 ± 2.02	-	-
Cy-dioxalyl glc *	593/594	581, 287	-	6.50 ± 0.56	-	-	-	-
*∑ Cy-deriv*			4.11	53.73	2.13	75.77	19.81	0.62
Dp-gal	465	303	5.02 ± 0.42	-	-	-	-	-
Dp-glc	465	303	0.81 ± 0.06	-	-	-	-	-
Dp-ara	435	303	0.63 ± 0.05	-	-	-	-	-
*∑ Dp-der*			6.46	-	-	-	-	-
Mv-gal	493	331	1.01 ± 0.09	-	-	-	-	-
Mv-glc	493	331	8.81 ± 0.78	-	-	-	-	-
Mv-ara	463	331	1.02 ± 0.09	-	-	-	-	-
*∑ Mv deriv*			10.84	-	-	-	-	-
Pg-glc	433	271	-	1.20 ± 0.09	-	-	-	1.29 ± 0.09
Pg-rut	579	433, 271	-	-	-	-	-	0.67 ± 0.06
Pg-3-acetyl-glc	475	271	-	-	-	-	-	0.49 ± 0.04
Pg-3mal-glc	519	271	-	-	-	-	-	0.35 ± 0.02
Pg-3,5diglc	595	433, 271	-	-	-	-	-	0.24 ± 0.02
Pg-3glc-rut	742/739		-	-	-	-	0.42 ± 0.03	-
E-(4,8)-Pg-glc	721	559	-	-	-	-	-	0.25 ± 0.02
(epi)afzelechin-Pg-glc	705	543, 407, 313	-	-	-	-	-	0.25 ± 0.02
CP Pg-glc	501	339	-	-	-	-	-	0.24 ± 0.02
*∑Pg deriv*			-	1.2	-	-	0.42	4.05
Pn-gal	463	301	0.65 ± 0.05	-	0.71 ± 0.06	-	-	-
Pn-glc	463	301	0.75 ± 0.06	0.82 ± 0.07	LOQ	-	-	-
Pn-ara	433	301	-	-	0.63 ± 0.05	-	-	-
*∑ Pn deriv*			1.40	0.82	1.34	-	-	-
Pt-gal	479	317	0.49 ± 0.03	-	-	-	-	-
Pt-glc	479	317	8.72 ± 0.72	-	-	-	-	-
Pt-ara	449	317	1.29 ± 0.09	-	-	-	-	-
*∑ Pt deriv*			10.50	-	-	-	-	-
ni	641	623, 505, 477, 605, 337	-	-	-	0.56 ± 0.04	-	-
total **			37.03	56.37	3.47	76.33	22.32	6.09

* or cyanidin 3-O-β-(6″-(3-hydroxy-3-methylglutaroyl)glucoside); ** of all peaks with max about 520 nm; Cy—cyanidin; Dp—delphinidin; Mv—malvidin; Pg—pelargonidin; Pn—peonidin; Pt—petunidin; gal—galactoside; glc—glucoside; ara—arabinoside, xyl—xyloside, rut—rutinoside, soph—sophoroside; sam—sambubioside; mal—malonyl, E—epicatechin; BB—bilberry wine; B—blackberry wine; C—cranberry wine; E—elderberry wine; R—raspberry wine; S—strawberry wine; LOQ—under limit of quantitation.

**Table 4:** Content of hydroxycinnamic acid derivatives in the investigated wines (mg/L).

	[M − H]^−^ *m*/*z*	MS^2^ *m*/*z*	BB	B	C	E	R	S
malonylo-CQA	439, 396	395, 219, 173, 295, 289	-	-	-	-	-	0.74 ± 0.05
neoChA	353	191, 179	-	6.42 ± 0.56	-	24.99 ± 2.08	-	-
CAH	341	197, 135, 161, 179	10.97 ± 0.93	LOQ	10.89 ± 0.87	9.98 ± 0.78	4.41 ± 0.37	-
pCoH	325	163, 145, 187, 265	-	54.35 * ± 4.89	-	-	12.35 * ± 0.96	6.31 ± 0.54
ChA	353	191, 179	LOQ	2.01 ± 0.17	5.59 ± 0.45	coeluted	-	-
CA	179	135	44.10 ± 3.99		8.67 ± 0.78	60.04 ± 5.33	15.32 ± 1.03	
FA	193	134	-	-	-	-	-	LOQ
p-CoA	163	119	14.77 ± 1.23	-	-	13.44 ± 1.02	-	4.60 ± 0.34
pCo der	411		2.27 ± 0.19	-	-	-	-	-
pCo der	525		-	-	-	19.86 ± 1.88	-	-
5-hydroxy F hex	371	281, 251, 221, 209	-	-	-	-	-	1.78 ± 0.16
ni	207		-	-	-	16.20 ± 1.52	-	-
total			73.48	63.95	37.06	150.79	32.18	13.43

- not identified in this wine; malonylo-CQA-malonylo-caffeoylquinic acid; neoChA-neochlorogenic acid; CAH—caffeic acid hexoside; ChA—chlorogenic acid; pCoH—p-coumaroylhexoside; CA—caffeic acid; FA—ferulic acid; pCoA—p-coumaric acid; pCoA der—p-coumaric acid derivative; 5-hydroxyFhex—5-hydroxyferuloyl hexose; tr—traces; BB—bilberry wine; B—blackberry wine; C—cranberry wine; E—elderberry wine; R—raspberry wine; S—strawberry wine; LOQ—under limit of quantitation; *—two peaks.

**Table 5:** Flavonols contents in the investigated wines (mg/L).

	[M − H]^−^ *m*/*z*	MS MS^2^ *m*/*z*	BB	B	C	E	R	S
								
M-glc	479	317	0.27 ± 0.02	-	-	-	-	-
M-ara	449	317	-	-	0.01 ± 0.00	-	-	-
M-xyl	449	317	-	-	0.02 ± 0.00	-	-	-
M-malonylglc	565	317	-	-	-	-	-	LOQ
M-dimethoxy-hex	507	344, 387	-	-	0.01 ± 0.00	-	-	-
M	317	179, 151, 192	0.28 ± 0.02	-	0.78 ± 0.06	-	-	-
∑ M derivatives			0.55	-	0.82	-	-	LOQ
Q-gal	463	301	-	0.36 ± 0.03	0.05 ± 0.00	0.10 ± 0.01	0.11 ± 0.01	-
Q-glc	463	301	0.17 ± 0.01	0.07 ± 0.01	-	2.36 ± 0.18	0.05 ± 0.00	0.19 ± 0.02
Q-ara	433	301	-	-	0.12 ± 0.01	-	-	-
Q-rut	609	301, 343, 463	LOQ	0.09 ± 0.00	-	5.04 ± 0.42	0.10 ± 0.01	-
Q-pent	433	301, 179, 151	-	LOQ	-	-	-	-
Q-xyl	433	301	0.05 ± 0.00	-	0.20 ± 0.02	-	-	-
Q-rha	447	301	-	-	0.25 ± 0.02	-	-	-
Q-gluc	477	301	-	0.31 ± 0.02	-	-	0.09 ± 0.01	0.38 ± 0.03
Q-diglc	625	283, 255, 463, 301	LOQ	-	-	-	-	-
Q-2gal-rha	609	283, 255, 300	-	-	-	-	0.09 ± 0.01	-
Q-3acetylhex	505	463, 301	-	0.39 ± 0.03	-	-	-	LOQ
Q-methoxyhex	493	463, 301	0.11 ± 0.01	-	-	-	-	-
Q3[6″(3hydroxy-3 methyl-glut)] gal	607	463, 301	-	0.51 ± 0.04	-	-	-	-
Q-malonyl-glc	549	503, 301	-	-	-	LOQ	-	-
methoxyQ-xyl	447	300	-	-	0.01 ± 0.00	-	-	-
Q-benzoyl gal	567	300	-	-	0.05 ± 0.00	-	-	-
Q	301	179, 151, 257	0.28 ± 0.02	0.07 ± 0.00	1.56 ± 0.14	1.35 ± 0.12	0.04 ± 0.00	0.05 ± 0.00
IsoQ	509	463	-	-	-	LOQ	-	-
dihydroQ glc	465	285, 151	-	LOQ	-	-	-	-
I=3-methylQ	315	631/632, 315	-	-	-	0.19 ± 0.02	-	-
∑ Q derivatives			0.63	1.80	2.24	9.04	0.48	0.62
K-gal	447	285	0.16 ± 0.01	-	-	-	0.10 ± 0.01	-
K-glc	447	285	0.16 ± 0.01	-	-	-	-	0.15 ± 0.01
K-rut	593	285	-	-	-	0.22 ± 0.02	-	-
K-pent	417	241, 152, 285	-	-	-	-	-	LOQ
K-gluc	461	415, 285	-	-	-	-	0.26 ± 0.02	0.15 ± 0.02
K	285	267	-	-	-	-	0.03 ± 0.00	-
dihydroK-glc	449	431, 287, 269, 259, 243, 179	-	LOQ	-	-	-	LOQ
dihydroK-rha	433	287	-	LOQ	-	-	-	-
K3(6″-*p*-Co)glc	593		-	LOQ	-	-	-	-
∑ K derivatives			0.16	-	-	0.22	0.39	0.30
total			1.43	1.81	3.02	9.27	0.87	0.92

- not identified in this wine; M—Myricetin; Q—Quercetin; K—Kaempferol; I—Isorhamnetin; gal—galactoside; glc—glucoside; ara—arabinoside, xyl—xyloside, rut—rutinoside, soph—sophoroside; rha—rhamnoside; glu—glucuronide; glut—glutaroyl; p-Co—p-coumaroyl; pent—pentoside; hex—hexoside; BB—bilberry wine; B—blackberry wine; C—cranberry wine; E—elderberry wine; R—raspberry wine; S—strawberry wine; LOQ—under limit of quantitation.

**Table 6:** Other compounds identified in investigated wines.

Tentative Compound	λmax	[M − H]^−^ *m*/*z*	MS^2^ *m*/*z*	BB	B	C	E	R	S
*acids*									
cinnamic acid	225	147	129, 85, 87, 103	+	+	+	+	+	+
vanillic acid	271	167		-	-	+	-	-	-
ascorbic acid	253	175	129, 115, 157, 85	+	+	+	-	+	-
shikimic acid	270	173	127, 83	+	+	-	+	+	+
p-hydroxybenzoic acid	277	137	93, 119, 110	-	-	+	+	+	+
benzoic acid	275	121	77, 121, 92	-	-	+	-	-	-
hydroxybenzoyl-glc	276, 309	299	137	-	-	-	-	-	+
protocatechuic acid	260, 294	153	109, 125, 83	+	+	+	+	-	-
protocatechuic acid hex		315	152, 108	-	+	+	-	-	+
1-O-protocatechuylhex		285	152, 108	-	-	+	-	-	+
sinapic acid hex	265, 382	385	339	-	-	-	+	-	-
brevifolin carboxylic acid	281	291	248, 247, 203	-	-	-	-	-	+
*cis*-ABA		263	153	+	+	-	-	-	-
*trans*-ABA		263	204	+	+	-	-	-	-
ABA-GE		425	263	-	+	-	-	-	-
ursolic acid=prunol		455	515	-	+	-	-	-	-
*Ellagic acid derivatives*									
ellagic acid	245, 278, 382	603 [2M] 301	467, 439, 179, 273, 257	+	+	-	+	+	+
ellagic acid pent	231	433	300/301	-	+	-	-	+	+
ellagic acid hex	255, 362	463	301	-	+	-	-	-	-
ellagic acid deoxyhex	231, 364	447	300/301, 257	-	-	-	-	-	+
dimethyl ellagic acid pent		461	300/301, 145	-	+	-	-	-	-
ellagic acid acetyl-ara	235, 273	475	301	-	-	-	-	+	-
methylellagic acid gluc	253, 361	491	315, 301, 257, 229	-	+	-	-	-	-
ellagic acid acetyl-methylpent	254, 364	489	301, 257, 229	-	+	-	-	-	-
ellagic acid rha		447	301	-	-	-	-	-	+
*Ellagitannins*									
ellagitannin	232, 270	679	664	-	-	-	-	-	+
HHDP glc		481	301, 275	-	+	-	-	-	+
galloyl-bis-HHDP glc		935	633, 301	-	+	-	-	-	-
galloyl-HHDP glc	280	633, 632.6	481, 301, 613, 301, 481, 783	-	+	-	-	-	+
bis-HDDP-glc	280	783	301, 481, 257, 229	-	+	-	-	-	+
tris-galloyl-HHDP hex		951	907, 783, 605, 301	-	+	-	-	-	+
davuriicin M1 (diHHDP-glc-galloyl-ellagic acid)		617[M-2H]^2−^, 1236	933, 631, 301	-	+	-	-	-	-
Sanguiin H-10 isomer (2)	232	[1567]^−^, [783]^2−^	935, 633, 301	-	+	-	-	+	-
Sanguiin H-2	245	1103, [551]^2^	935, 633, 469, 301	-	-	-	-	+	-
castalagin/vescalagin		933	301	-	+	-	-	-	-
pedunculagin/sanguin isomer H10	268, 377	783	633, 301, 1266, 934, 1104	-	+	-	-	+	-
Sanguin H6	340, 352, 366	935/934 [M-2H]^2−^ 1870	633, 301, 897, 916, 783, 1567, 1235, 633, 301	-	+	-	-	+	-
*Gallic acid derivatives*									
gallic acid	286	169	125	+	-	-	-	-	+
methyl gallate		183/184		-	-	+	-	-	-
galloylquinic acid		343	191, 169	-	-	-	-	-	+
gallic acid deriv	280, 451	635	483	+	-	-	-	-	-
*Procyanidins*									
epigallocatechin	283	611		-	+	-	-	-	+
gallocatechin		306/305		-	+	-	-	-	-
catechin	280	289	245, 205, 179	+	+	+	-	-	+
epicatechin	285	289	245, 205, 271, 179	-	+	-	-	+	-
Procyanidin dimer	277	575	490, 499, 413	-	-	+	-	+	-
ni	277	575	377, 395, 333, 273, 1007	-	-	+	-	-	-
	277	575	863/864, 499, 413, 267, 289, 699, 1025	-	-	+	-	-	-
	277	575	499, 490, 861, 423, 289, 999, 1025	-	-	+	-	-	-
	277	575	395, 351, 371, 289, 1025	-	-	+	-	-	-
	277	575	423, 449, 539, 285, 557, 1025	-	-	+	-	-	-
B type dimer (procyanidin dimer)	282	577	425, 407, 451	+	-	-	-	-	+
Procyanidin B1	278	577	425, 407	-	+	+	-	-	+
		577	397, 373, 273, 415, 1019	-	-	+	-	-	-
Procyanidin trimer (Atype)	280	863	711, 411, 559, 693	-	-	+	-	-	-
Procyanidin trimer (Btype)	281	865	695, 577, 407, 847	-	-	+	-	-	+
Procyanidin tetramer (Btype)	276	1152/1153		-	-	+	-	-	-
dimer (Cat-Afz) propelargonidin dimer	279	561	289, 543, 435	-	+	-	-	-	+
trimer A type	276	863	711, 693, 411, 459, 559, 289	-	-	+	-	-	-
ni		863	575, 711, 693, 559, 285, 1601	-	-	+	-	-	-
Trimer (Cat-Cat-Afz)		849		-	-	-	-	-	+
Flavone.									
apigenin pent		401	269, 161	-	+	-	-	-	-
apigenin glc		431	370, 269, 311	-	+	-	-	-	-
*Biflavonoids*									
pentahydroxyflavan dimer	250	579	271, 289	-	-	-	-	+	-
tetrahydroxyflavan–pentahydroxyflavan dimer		563	273, 291, 411, 427	-	-	-	-	+	-
*Stilbenoids*									
trans-resveratrol-glc		389	185, 227	+	+	-	-	-	-
*Unknown compounds*									
ni		340	294, 188, 161	-	-	-	+	-	-
ni	226, 278, 397	405	225	-	-	-	+	-	-
ni	259	391	217, 373, 111, 216, 191	-	-	-	-	+	-
ni	226, 284	379	241	-	-	-	+	-	-
ni	281	333	165, 289, 183	-	-	+	-	-	-

ni—not identified compound; +—present; -—absent; glc—glucoside, hex—hexoside, ABA—abscisic acid; ABA-GE—abscisic acid D-glucopyranosyl ester; BB—bilberry wine; B—blackberry wine; C—cranberry wine; E—elderberry wine; R—raspberry wine; S—strawberry wine.

**Table 7:** Inhibition zones (mm).

	*Escherichia coli*	*Salmonella* Enteritidis	*Bacillus cereus*	*Listeria monocytogenes*	*Staphylococcus aureus*	*Candida albicans*
Bilberry	-	-	1.73 ± 0.12 ^A^	-	-	-
Blackberry	-	-	2.00 ± 0.25 ^bA^	1.00 ± 0.00 ^aA^	-	-
Cranberry	-	-	1.83 ± 0.23 ^aA^	2.33 ± 0.58 ^aB^	-	-
Elderberry	-	-	-	-	-	-
Raspberry	-	-	4.00 ± 0.71 ^B^	-	-	-
Strawberry	2.67 ± 0.58 ^a^	-	1.83 ± 0.23 ^aA^	-	-	-

- No inhibitory effect; a,b—Different letters indicate a significant difference in rows (*p* < 0.05); A,B—Different letters indicate a significant difference in columns (*p* < 0.05).

## Supplementary Materials

The following are available online at https://www.mdpi.com/2304-8158/9/12/1783/s1, Figure S1: Chromatogram at 520 nm of bilberry wine obtained with HPLC-DAD; 3-Dp-gal; 4-Dp-glc; 5-Cy-gal; 6-Dp-ara; 7-Cy-glc; 8-Pt-gal; 9-Pt-glc; 10-Pn-gal;12-Pt-ara;13-Pn-glc; 14-Mv-gal; 15-Mv-glc; 16-Mv-ara. Figure S2: Chromatogram at 320 nm of bilberry wine obtained with HPLC-DAD; 7-CAH; 8-CA; 10-p-CoA; 18-p-CoA der. Figure S3: Chromatogram at 360 nm of bilberry wine obtained with HPLC-DAD; 11-M-glc; 14-Q-glc; 15-Q-metoxyhex; 18-K-gal; 19-K-glc; 21-M; 22-Q. Figure S4: Chromatogram at 520 nm of blackberry wine obtained with HPLC-DAD; 1-Cy-gal; 2-Cy-glc; 3-Cy-xyl; 5-Pg-glc; 7-Cy-3mal-glc; 8-Cy-6mal-glc; 9-Cy-dioxalyl glc. Figure S5: Chromatogram at 320 nm of blackberry wine obtained with HPLC-DAD; 11-CAH; 15-neoChA; 18-ChA; 21-pCoH. Figure S6: Chromatogram at 360 nm of blackberry wine obtained with HPLC-DAD; 23-Q-rut; 24-Q-gal; 25-Q-gluc; 26-Q-glc; 28-Qacetylhex; 29-Q-3[6″ (3hydroxy-3 methyl-glut)] gal. Figure S7: Chromatogram at 520 nm of cranberry wine obtained with HPLC-DAD; 10-Cy-gal; 11-Cy-glc; 14-Cy-ara; 15-Pn-gal; 17-Pn-glc; 18-Pn-ara. Figure S8: Chromatogram at 320 nm of cranberry wine obtained with HPLC-DAD; 11-CAH; 12-ChA; 13-CA. Figure S9: Chromatogram at 360 nm of cranberry wine obtained with HPLC-DAD; 7-M-xyl; 9-M-ara; 14-Q-gal; 17-M-dimetoxy-hex; 18-Q-xyl; 19-Q-ara; 20-Q-rha; 21-M; 22-metoxyQ-xyl; 23-Q; 24-Q-benzoyl gal. Figure S10: Chromatogram at 520 nm of elderberry wine obtained with HPLC-DAD; 3-Cy-sam-5-glc; 4-Cy-sam; 5-Cy-glc. Figure S11: Chromatogram at 320 nm of elderberry wine obtained with HPLC-DAD; 7-neoChA; 13-CAH; 16-CA/ChA -coeluted; 23-p-CoA der; 32-ni (λmax = 323). Figure S12: Chromatogram at 360 nm of elderberry wine obtained with HPLC-DAD; 17-Q-rut; 18-Q-glc;19-K-rut; 20-3-methylQ; 21-Q. Figure S13: Chromatogram at 520 nm of raspberry wine obtained with HPLC-DAD; 5-Cy-soph; 6-Cy-3(2glc) rut; 7-Pg-3glc-rut/Cy-glc. Figure S14: Chromatogram at 320 nm of raspberry wine obtained with HPLC-DAD; 12-CAH; 16, 17-pCoAHs; 18-CA. Figure S15 Chromatogram at 360 nm of raspberry wine obtained with HPLC-DAD; 14-Q-2gal-rha; 20-Q-rut; 21- Q-gluc; 22-K-gal; 23-K-gluc; 25-Q; 26-K. Figure S16: Chromatogram at 520 nm of strawberry wine obtained with HPLC-DAD; 7-Cy-gal; 12 Cy-glc; 15- Pg-glc; 16-Pg-rut; 17-Pg-3,5diglc; 18-Pg-3mal-glc; 22-Pg-3-acet-glc. Figure S17: Chromatogram at 320 nm of strawberry wine obtained with HPLC-DAD; 12-malonyloCQA; 19-p-CoH; 31-pCoA; 42-5-hydroxyF hex. Figure S18 Chromatogram at 360 nm of strawberry wine obtained with HPLC-DAD; 39-Q-gluc; 40-Q-glc; 41-K-gluc; 42-Q.
