# Supplementary material for: Characterization of Phytochemicals in Berry Fruit Wines Analyzed by Liquid Chromatography Coupled to Photodiode-Array Detection and Electrospray Ionization/Ion Trap Mass Spectrometry (LC-DAD-ESI-MSn) and Their Antioxidant and Antimicrobial Activity
Source: Foods. 2020 Dec 1;9(12):1783. doi: 10.3390/foods9121783 (PMC7761082; doi:10.3390/foods9121783)
Supplement: Supplementary file 1 [file foods-09-01783-s001.pdf]

Bilberry

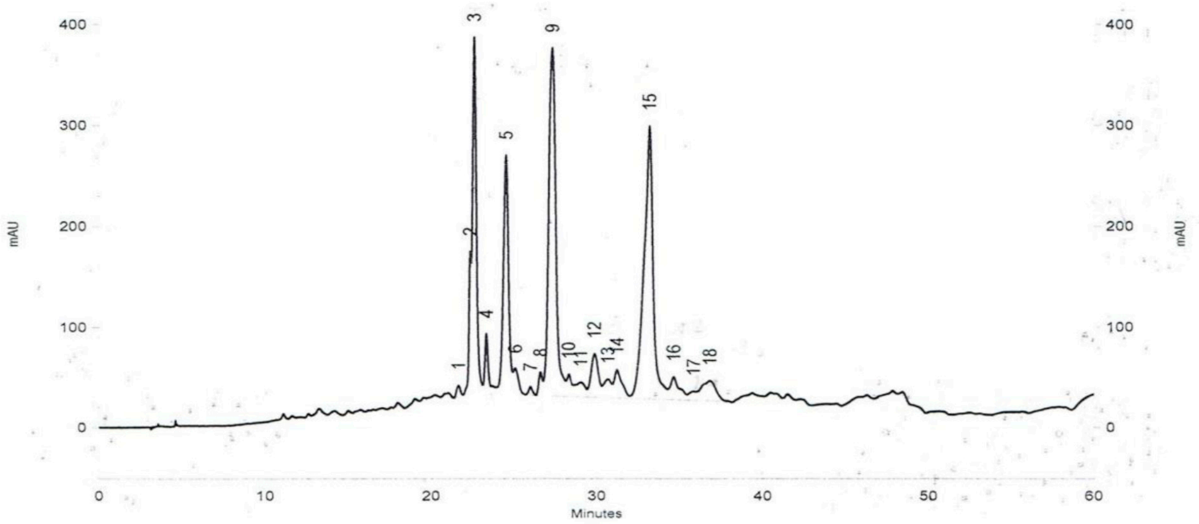

Figure S1. Chromatogram at 520 nm of bilberry wine obtained with HPLC-DAD; 3-Dp-gal; 4-Dp-glc; 5-Cy-gal; 6-Dp-ara; 7-Cy-glc; 8-Pt-gal; 9-Pt-glc; 10-Pn-gal;12-Pt-ara;13-Pn-glc; 14-Mv-gal; 15-Mv-glc; 16-Mv-ara.

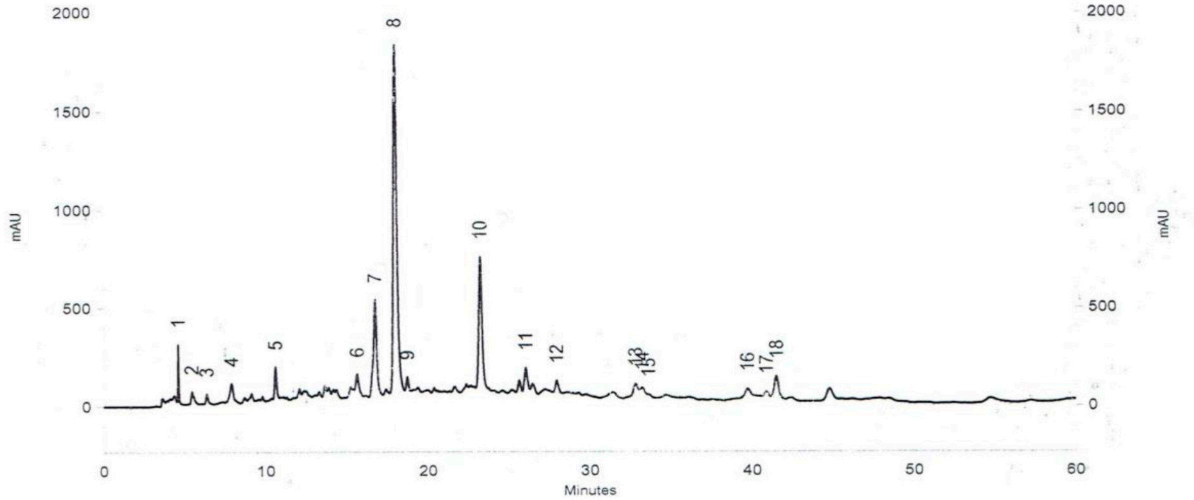

Figure S2. Chromatogram at 320 nm of bilberry wine obtained with HPLC-DAD; 7-CAH; 8-CA; 10-*p*-CoA; 18-*p*-CoA der.

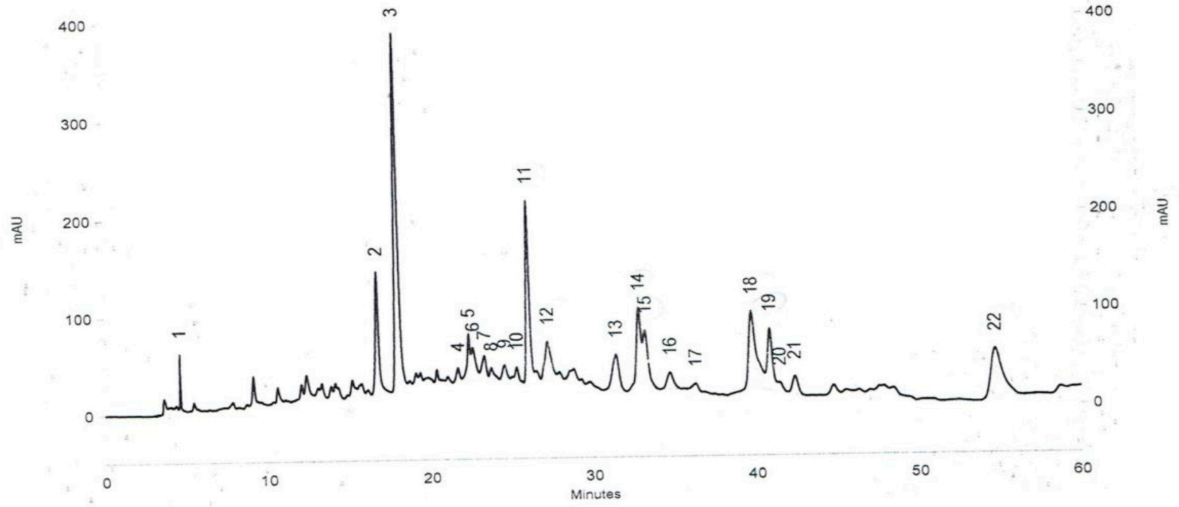

Figure S3. Chromatogram at 360 nm of bilberry wine obtained with HPLC-DAD; 11-M-glc; 14-Q-glc; 15-Q-metoxyhex; 18-K-gal; 19-K-glc; 21-M; 22-Q.

Blackberry wine

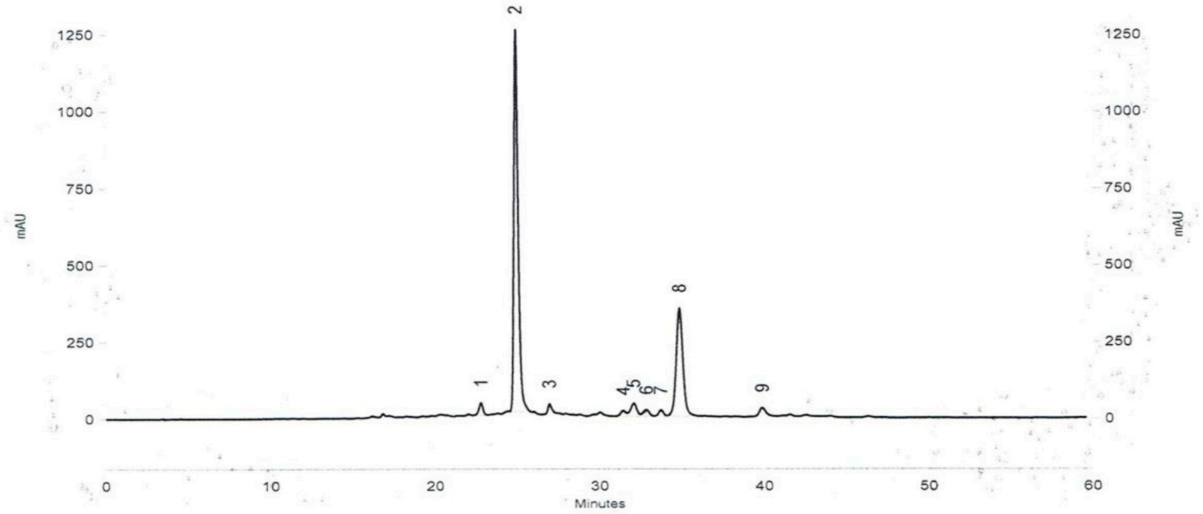

Figure S4. Chromatogram at 520 nm of blackberry wine obtained with HPLC-DAD; 1-Cy-gal; 2-Cy-glc; 3-Cy-xyl; 5-Pg-glc; 7-Cy-3mal-glc; 8-Cy-6mal-glc; 9-Cy-dioxalyl glc.

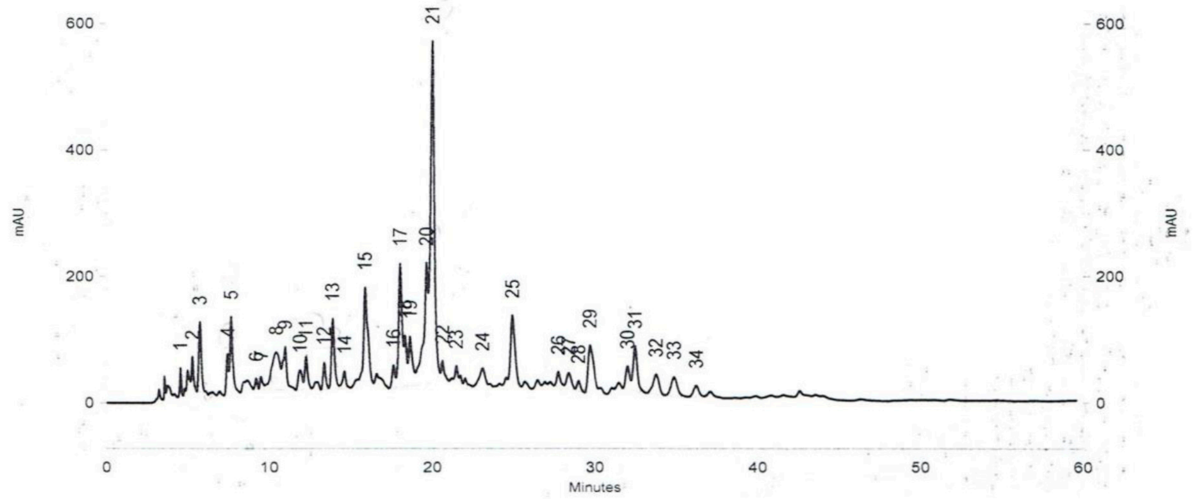

Figure S5. Chromatogram at 320 nm of blackberry wine obtained with HPLC-DAD; 11-CAH; 15-neoChA; 18-ChA; 21-pCoH.

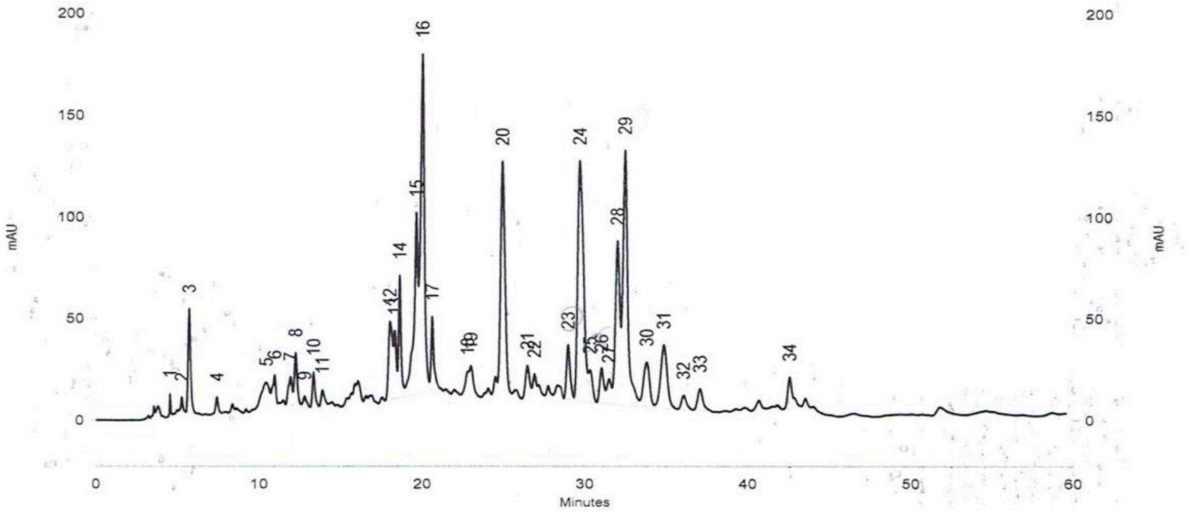

Figure S6. Chromatogram at 360 nm of blackberry wine obtained with HPLC-DAD; 23-Q-rut; 24-Q-gal; 25-Q-gluc; 26-Q-glc; 28-Qacetylhex; 29-Q-3[6″ (3hydroxy-3 methyl-glut)] gal.

Cranberry wine

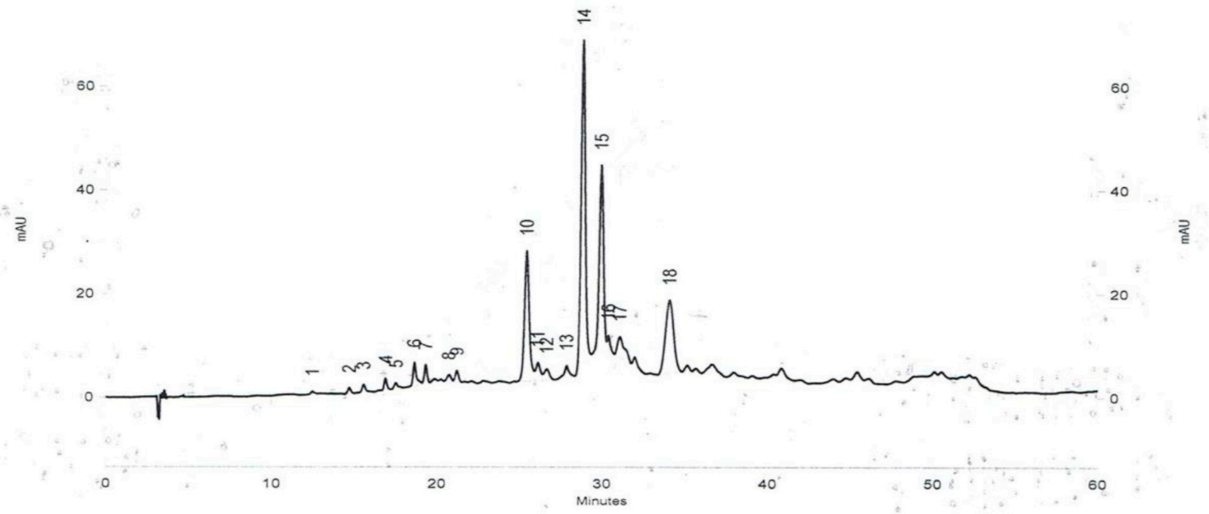

Figure S7. Chromatogram at 520 nm of cranberry wine obtained with HPLC-DAD; 10-Cy-gal; 11-Cy-glc; 14-Cy-ara; 15-Pn-gal; 17-Pn-glc; 18-Pn-ara.

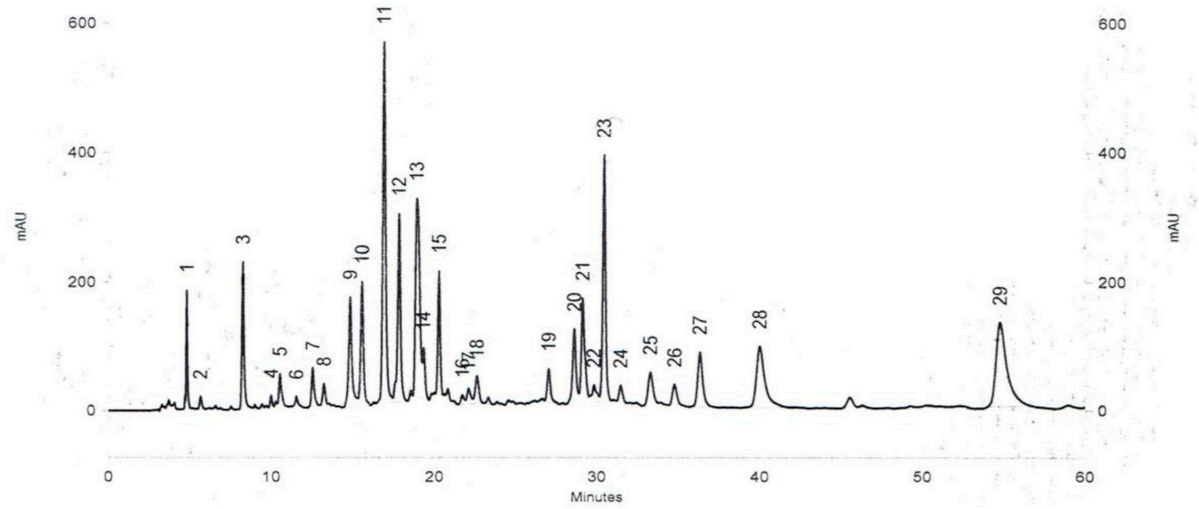

Figure S8. Chromatogram at 320 nm of cranberry wine obtained with HPLC-DAD; 11-CAH; 12-ChA; 13-CA.

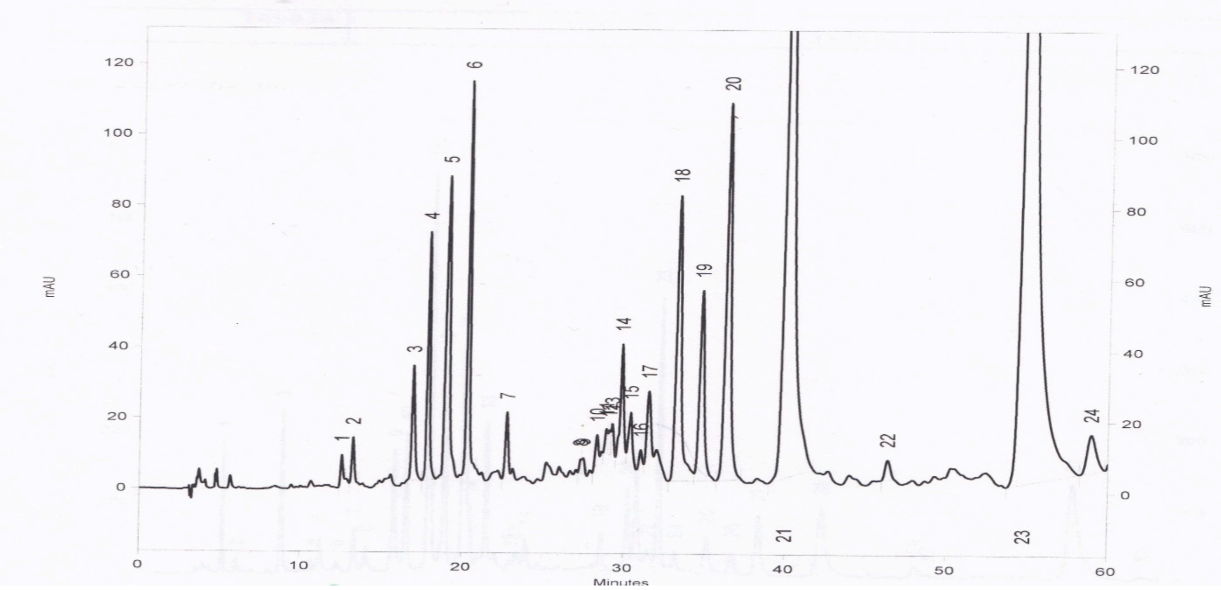

Figure S9. Chromatogram at 360 nm of cranberry wine obtained with HPLC-DAD; 7-M-xyl; 9-M-ara; 14-Q-gal; 17-M-dimetoxy-hex ;18-Q-xyl; 19-Q-ara; 20-Q-rha; 21-M; 22-metoxyQ-xyl; 23-Q; 24-Q-benzoyl gal.

Elderberry wine

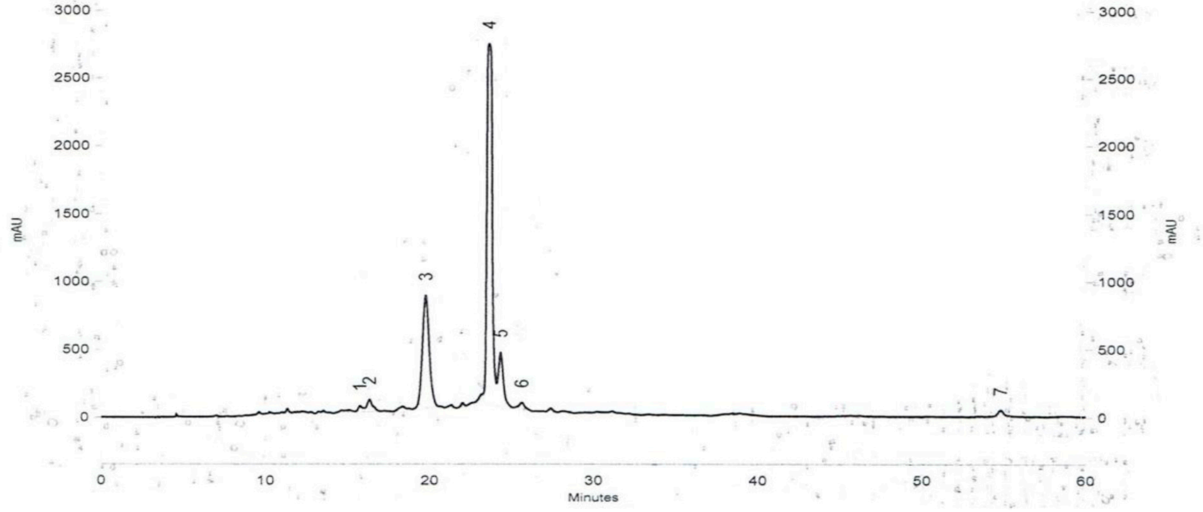

Figure S10. Chromatogram at 520 nm of elderberry wine obtained with HPLC-DAD; 3-Cy-sam-5-glc; 4-Cy-sam; 5-Cy-glc.

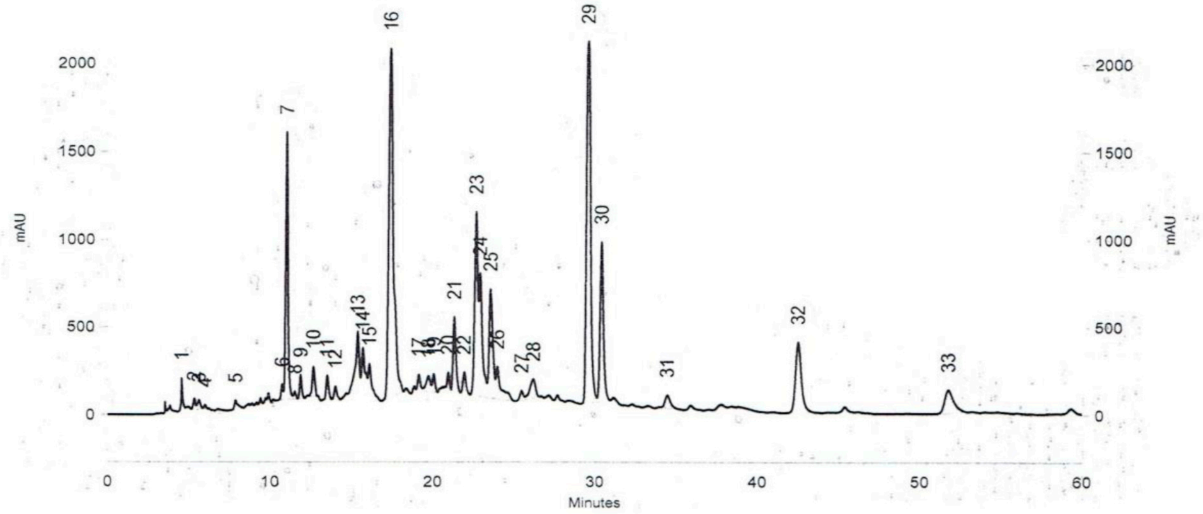

Figure S11. Chromatogram at 320 nm of elderberry wine obtained with HPLC-DAD; 7-neoChA; 13-CAH; 16-CA/ChA -coeluted; 23-p-CoA der; 32-ni (λmax=323).

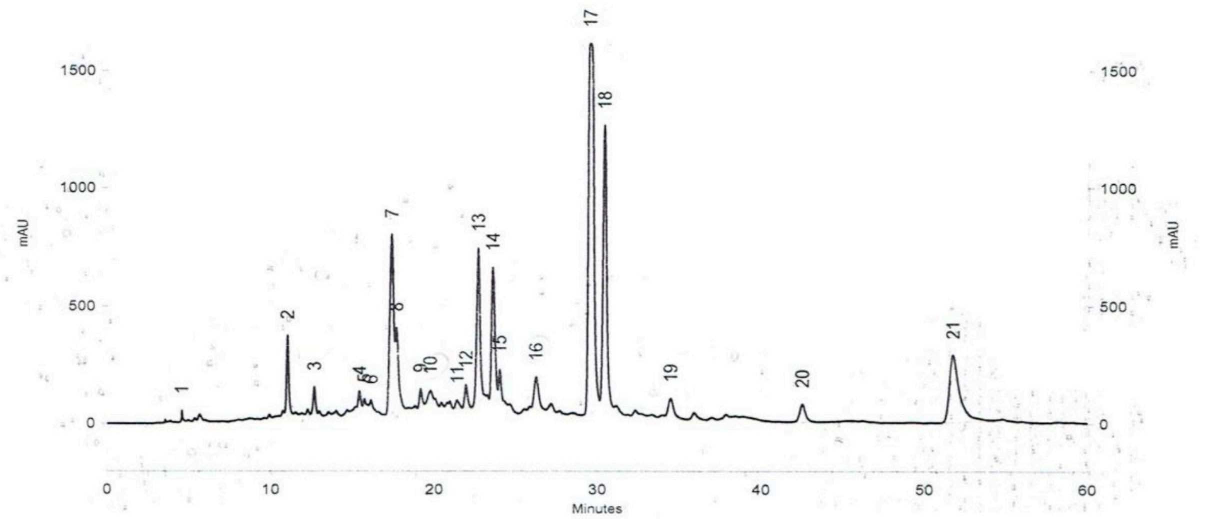

Figure S12. Chromatogram at 360 nm of elderberry wine obtained with HPLC-DAD; 17-Q-rut; 18-Q-glc;19-K-rut; 20-3-methylQ; 21-Q.

Raspberry wine

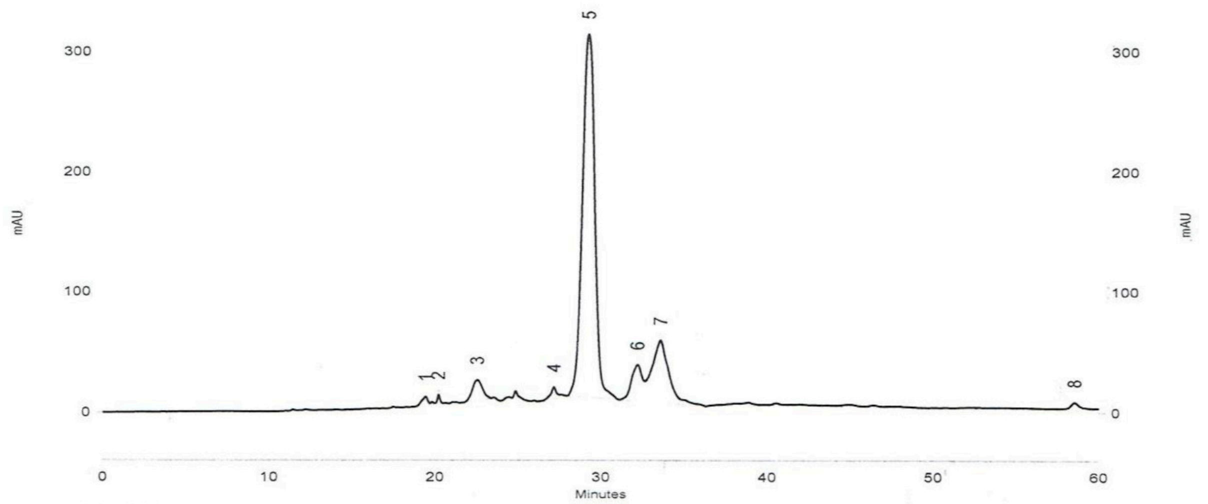

Figure S13. Chromatogram at 520 nm of raspberry wine obtained with HPLC-DAD; 5-Cy-soph; 6-Cy-3(2glc) rut; 7-Pg-3glc-rut/Cy-glc.

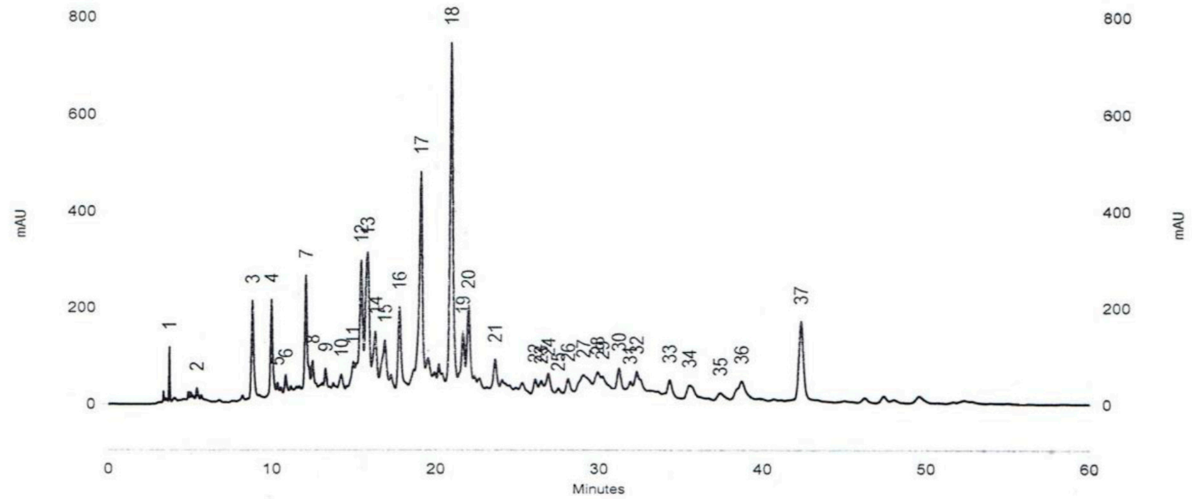

Figure S14. Chromatogram at 320 nm of raspberry wine obtained with HPLC-DAD; 12-CAH; 16, 17-pCoAHs; 18-CA.

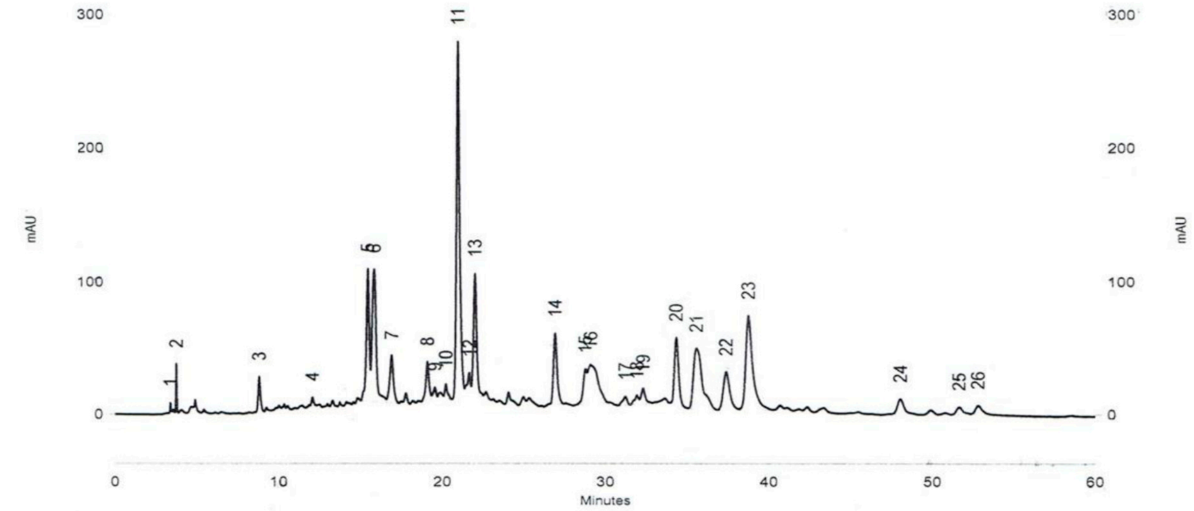

Figure S15 Chromatogram at 360 nm of raspberry wine obtained with HPLC-DAD; 14-Q-2gal-rha; 20-Q-rut; 21-Q-gluc; 22-K-gal; 23-K-gluc; 25-Q; 26-K.

Strawberry wine

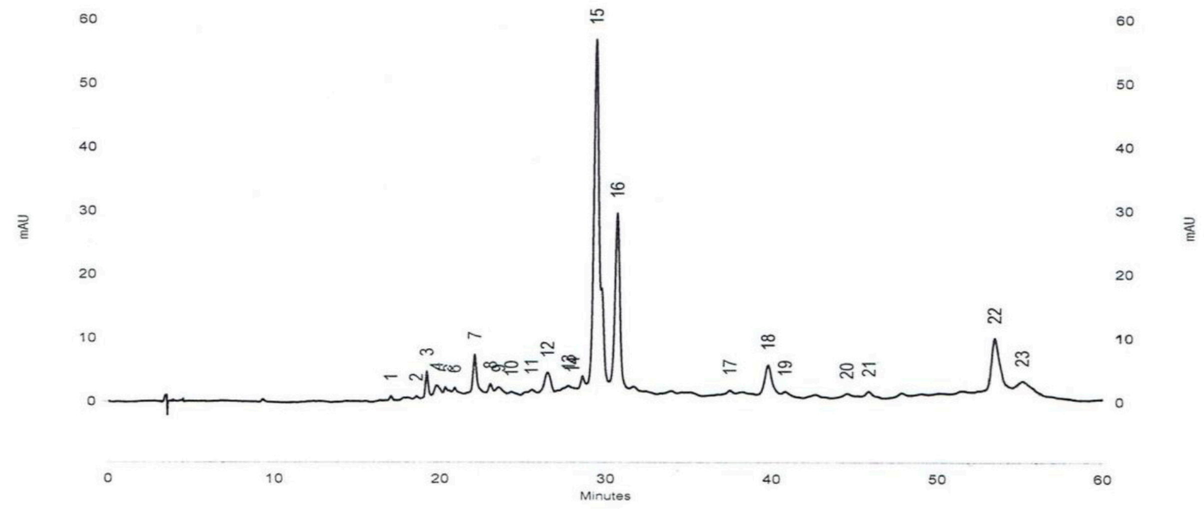

Figure S16. Chromatogram at 520 nm of strawberry wine obtained with HPLC-DAD; 7-Cy-gal; 12 Cy-glc; 15- Pg-glc; 16-Pg-rut; 17-Pg-3,5diglc; 18-Pg-3mal-glc; 22-Pg-3-acet-glc.

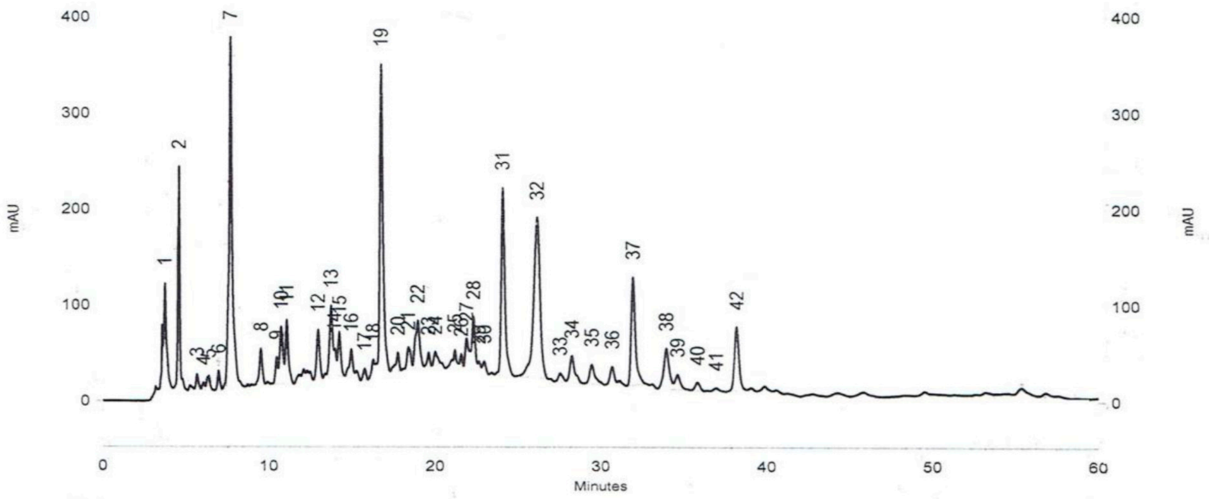

Figure S17. Chromatogram at 320 nm of strawberry wine obtained with HPLC-DAD; 12-malonyloCQA; 19-p-CoH; 31-pCoA; 42-5-hydroxyF hex.

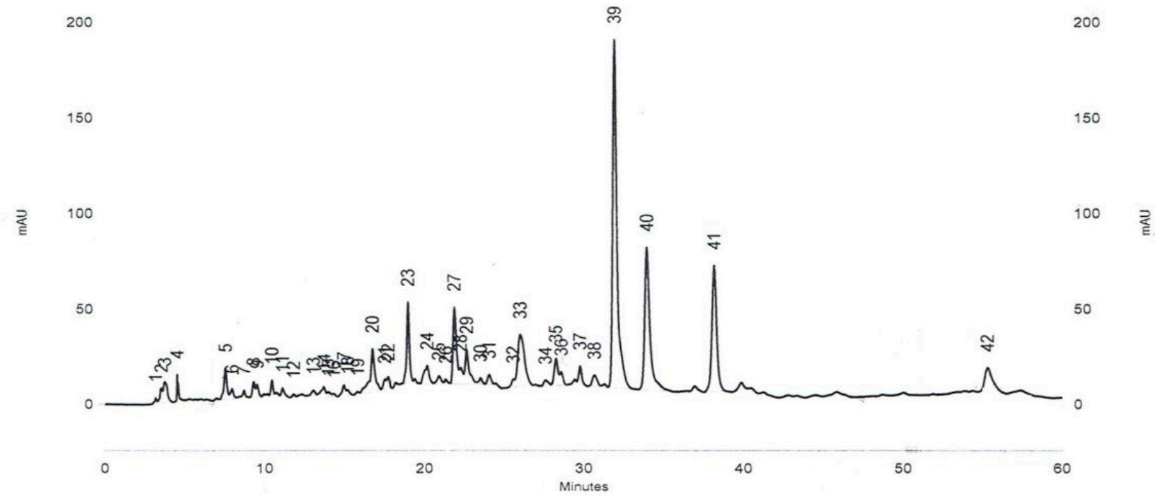

Figure S18 Chromatogram at 360 nm of strawberry wine obtained with HPLC-DAD; 39-Q-gluc; 40-Q-glc; 41-K-gluc; 42-Q.
